# Nirmatrelvir/ritonavir for patients with SARS-CoV-2 infection and impaired kidney function during the Omicron surge

**DOI:** 10.3389/fphar.2023.1147980

**Published:** 2023-03-22

**Authors:** Jiayi Yan, Hong Cai, Jieying Wang, Mingli Zhu, Ping Li, Peiying Li, Bin Wu, Xiajing Che, Leyi Gu, Shan Mou

**Affiliations:** ^1^ Department of Nephrology, Molecular Cell Lab for Kidney Disease, Shanghai Peritoneal Dialysis Research Center, Ren Ji Hospital, Uremia Diagnosis and Treatment Center, Shanghai Jiao Tong University School of Medicine, Shanghai, China; ^2^ Academy of Integrative Medicine, Shanghai University of Traditional Chinese Medicine, Shanghai, China; ^3^ Clinical Research Center, Ren Ji Hospital, School of Medicine, Shanghai Jiao Tong University, Shanghai, China; ^4^ Department of Critical Care Medicine, Ren Ji Hospital, School of Medicine, Shanghai Jiao Tong University, Shanghai, China

**Keywords:** nirmatrelvir/ritonavir, COVID-19, SARS-CoV-2, omicron, outcomes, impaired kidney function

## Abstract

**Background:** Nirmatrelvir/ritonavir has demonstrated effectiveness in high-risk patients with coronavirus disease 2019 (COVID-19). However, investigations on the efficacy and safety of nirmatrelvir/ritonavir in patients with kidney dysfunction are limited.

**Methods:** Data were collected from the patients admitted to a COVID-19 referral center in Shanghai, China. Patients were at least 18 years of age and had a baseline estimated glomerular filtration rate (eGFR) of <60 ml/min/1·73 m^2^. The primary endpoint was a composite of all-cause mortality, intensive care unit admission, or cardiovascular events. The secondary endpoint was viral shedding.

**Results:** Among the 195 participants, 73 received nirmatrelvir/ritonavir. A lower risk of the primary endpoint was observed in nirmatrelvir/ritonavir recipients compared with non-recipients [adjusted HR 0.56 (95% CI: 0.32–0.96); *p =* 0.035]. Nirmatrelvir/ritonavir recipients experienced a shorter duration of viral shedding [adjusted HR 3·70 (95%CI: 2.60–5.28); *p* < 0.001) and faster viral load clearance versus non-recipients. Among the nirmatrelvir/ritonavir users, earlier initiation of nirmatrelvir/ritonavir within 5 days since COVID-19 diagnosis was related with shorter viral shedding time (adjusted HR 7.84 [95% CI: 3.28–18.76]; *p* < 0.001) compared to late initiation. No patients reported serious adverse events during treatment.

**Conclusion:** Our findings support the early initiation of nirmatrelvir/ritonavir for high-risk patients with impaired kidney function. This could improve patient outcomes and shorten the viral shedding period.

## 1 Introduction

Coronavirus disease 2019 (COVID-19), originated from severe acute respiratory syndrome coronavirus 2 (SARS-CoV-2), has become a global pandemic (Hu et al., 2021). SARS-CoV-2 variants have also emerged within the last few years. At present, the Omicron variant is the predominant strain (Graham, 2021; Fan et al., 2022; Gao et al., 2022). Data suggests that the Omicron variant is less virulent than the preceding ones (Christensen et al., 2022). However, the vulnerable individuals, such as the elderly and those with comorbidities, have worse outcomes than the general population (Kim et al., 2021; Zhang et al., 2022a; Lu et al., 2022). The Omicron variant evades the present neutralizing antibodies and increases the risk of vaccine breakthrough associated with the mutation ability of the spike protein (Christensen et al., 2022; Dejnirattisai et al., 2022). The immune escape character of the Omicron variant may challenge the development of new medications and vaccines. In addition, studies show that individuals with coexisting kidney dysfunction are vulnerable to more severe COVID-19-related illnesses and poorer prognosis than those with normal kidney function. Patients with concomitant kidney dysfunction are among the vulnerable population requiring specific care and management (Ozturk et al., 2020; Carlson et al., 2021; Flythe et al., 2021; Russo et al., 2021).

The new antiviral candidate, Paxlovid (nirmatrelvir/ritonavir, a SARS-CoV-2 protease inhibitor), is emergently authorized for the treatment of patients with COVID-19 (Food and Drug Administration, 2021). Ritonavir acts as a CYP3A4 inhibitor enhancing the bioavailability of nirmatrelvir. Results from the Evaluation of Protease Inhibition for COVID-19 in High-Risk Patients (EPIC-HR) study in unvaccinated individuals demonstrate the effectiveness of nirmatrelvir/ritonavir to reduce the risk of death or hospitalization by 89%, when compared with a placebo (Mahase, 2021; Hammond et al., 2022). The efficacy of SARS-CoV-2 medications probably change along with the evolution of new virus variants. Nirmatrelvir/ritonavir targets Mpro, which is the essential protein of SARS-CoV-2. Given the well-conserved characteristics of the Mpro active site, an Mpro inhibitor may be able to maintain anti-viral activity against potentially immune-evasive SARS-CoV-2 variants (Owen et al., 2021; Qiao et al., 2021). In the EPIC-HR trial, adverse events that required emergency management were comparable between nirmatrelvir/ritonavir (19%) and placebo (21%), most of which were mild in intensity (Hammond et al., 2022). Certain medications ought to be prescribed with caution or avoided in patients with an impaired kidney function. To date, nirmatrelvir/ritonavir is not yet recommended in patients with severely impaired kidney function as there is insufficient evidence for nirmatrelvir/ritonavir application in this patient population (Fact Sheet for Healthcare Providers, 2022).

It is worth noting that, after genomic analysis of the emergent viruses in Shanghai, China, in March 2022, the new infection cases were revealed to be caused by viruses belong to the Omicron BA.2.2 sub-lineages (Zhang et al., 2022b). Data has demonstrated that nirmatrelvir/ritonavir is effective against the Delta and Omicron variants. However, data are limited concerning nirmatrelvir/ritonavir’s effectiveness on subsequent Omicron sub-lineages. To address these knowledge gaps mentioned above, we examined the efficacy and safety of nirmatrelvir/ritonavir therapy in patients with Omicron variant infections and impaired kidney function.

## 2 Patients and methods

### 2.1 Study subjects

City-wide management and control were implemented in Shanghai during the Omicron surge since March 2022. Daily screening for SARS-CoV-2 infection was performed and individuals who were confirmed with positive real-time polymerase chain reaction (RT-PCR) findings of SARS-CoV-2 were then transferred to COVID-19 referral centers for further treatment (Zhang et al., 2022b). We conducted a retrospective cohort study in one of the COVID-19 referral centers, from 1 April to 30 June 2022. Patients were included if they were 18 years or older, with a baseline estimated glomerular filtration rate (eGFR) of <60 ml/min/1·73 m^2^, including maintenance hemodialysis patients (Levey et al., 2009). We excluded patients with systematic antiviral medication use either before or during admission. Follow-up ended at the time points: patient death, the occurrence of endpoints, or the end of the study, whichever came first. The study was carried out in accordance with Declaration of Helsinki and approved by the institution ethics committee (KY 2022-159-B). The requirement of informed consent was remitted due to the retrospective study design.

### 2.2 Data collection

The COVID-19 registry of the referral center was established during the Omicron surge. Patient information was recorded carefully and verified by specific data administrators. Our research data were extracted from the registry, including demographic and clinical information, such as age, sex, laboratory findings, coexisting chronic diseases, medications, and clinical outcomes. COVID-19 related data included symptoms, clinical presentation, nirmatrelvir/ritonavir therapy, and the duration of viral shedding. We found no missing of key variables after examination of the retrieved research data.

### 2.3 Exposure

Patients who received nirmatrelvir/ritonavir therapy during hospitalization were defined as having treatment exposure. Prescription and dose adjustment of nirmatrelvir/ritonavir was on the basis of the medication instructions and pharmacist consultation as there was limited data in patients with severely impaired kidney function. Eligibility for the administration of nirmatrelvir/ritonavir considered contraindications and drug-drug interactions, which were described in the medication instructions (Fact Sheet for Healthcare Providers, 2022). According to the treatment recommendations, nirmatrelvir/ritonavir was administered as 150 mg of nirmatrelvir with 100 mg of ritonavir, twice daily for 5 days for those with an eGFR of 30 to <60 ml/min/1·73 m^2^. For patients with advanced stage kidney dysfunction of eGFR <30 ml/min/1·73 m^2^ not receiving maintenance hemodialysis, we cautiously applied the dosage as 150 mg of nirmatrelvir with 100 mg of ritonavir, once daily for 5 days, given the unavailability of data on nirmatrelvir/ritonavir use in this patient population. All the recruited maintenance hemodialysis patients received routine dialysis treatment thrice a week (4 h per session). Blood flow rate was 220–280 ml/min, and dialysate flow rate was 500 ml/min. The mean Kt/V (dialysis adequacy) was 1·67. These patients received a dosage of 150 mg of nirmatrelvir with 100 mg of ritonavir, once daily for 5 days (after the dialysis session if on the dialysis day). All of the patients receiving nirmatrelvir/ritonavir were informed prior to the trial and provided their relevant informed consent. The protocol was additionally approved by the medical service department of the hospital.

### 2.4 Outcomes

The primary endpoint was a composite of all-cause mortality, intensive care unit (ICU) admission, or cardiovascular events. The secondary endpoint was viral nucleic acid shedding, which was defined according to the National guidelines (National Health Commission of the People’s Republic of China, 2022), including: 1) Cycle threshold (Ct) value of 35 or higher in both ORF1ab and N genes on RT-PCR assay for SARS-CoV-2; 2) two consecutive negative nucleic acid results of at least 24 h apart. The index date of nirmatrelvir/ritonavir recipients corresponded to the day the recipient initiated the nirmatrelvir/ritonavir treatment, while the index date corresponded to the day of confirmed diagnosis of SARS-CoV-2 infection for non-nirmatrelvir/ritonavir recipients. When analyzing the viral shedding time in the subgroup of patients who received nirmatrelvir/ritonavir treatment, the index date corresponded to the day of confirmed diagnosis of SARS-CoV-2 infection. The duration of viral shedding was defined as the time interval from the respective index date to the time point of viral shedding.

### 2.5 Safety analysis

We considered the safety endpoint as the adverse events after nirmatrelvir/ritonavir uptake. We compared the results of several essential clinical parameters at baseline and 10 days after nirmatrelvir/ritonavir initiation and collected the adverse events, serious adverse events, and withdrawal of nirmatrelvir/ritonavir treatment due to adverse events. Safety information were collected from nirmatrelvir/ritonavir initiation to the end of the study or patient death.

### 2.6 Statistical analysis

Continuous variables were presented as median (interquartile range) or mean (Standard Deviation) and compared with Mann-Whitney U test or *t*-test. Categorical variables were presented as numbers (%) and compared by χ^2^ test. Kaplan-Meier analysis with a log-rank test and multivariate Cox proportional-hazards regression model were used to estimate the association between nirmatrelvir/ritonavir treatment and the outcomes. Nirmatrelvir/ritonavir therapy was treated as a time-dependent variable in the survival analyses, allowing subjects to convert from one exposure group to another. SPSS software, version 26 (IBM Corp., Armonk, NY, United States) and R statistical software, version 3.5.0 (R Foundation) were used for statistical analyses. A two-sided *p* < 0·05 was considered statistically significant.

## 3 Results

### 3.1 Characteristics of the patients at baseline

A total of 3,310 patients were admitted with SARS-CoV-2 infection during the study period, 195 of whom met the eligibility criteria. The evaluation process for eligibility is described in Supplementary Figure S1. Among all the patients enrolled, the median age was 74.0 years and 109 (55.9%) participants were male. The participants were divided according to the baseline eGFR level. Among these participants, 73 (37.4%) received nirmatrelvir/ritonavir therapy at a median age of 77·0 years. Among these recipients, 45 (48.4%) had an eGFR of 30–59 ml/min/1·73 m^2^, nine (22·5%) had an eGFR<30 ml/min/1·73 m^2^ and were not receiving dialysis, and 19 (30.6%) were receiving maintenance hemodialysis. The most common concomitant comorbidities were hypertension and cardiovascular disease. 132 (67·7%) patients had two or more coexisting conditions (Table 1).

**TABLE 1:** Baseline characteristics of patients with SARS-CoV-2 infection and impaired kidney function, according to eGFR group.

	Total (*n* = 195)	30 ≤ eGFR<60 (ml/min/1·73 m^2^) (*n* = 93)	eGFR<30 (ml/min/1·73 m^2^) (Non-dialysis) (*n* = 40)	Maintenance hemodialysis (*n* = 62)	*p*
Age (year)	74.00 (64.00, 85.00)	80.00 (70.00, 88.00)	80.00 (69.00, 89.00)	64.50 (55.00, 73.00)	<0.001
Sex Male, n (%)	109 (55.9)	48 (51.6)	19 (47.5)	42 (67.7)	0.068
BMI (kg/m^2^)	21.30 (18.75, 24.25)	20.94 (18.80, 23.48)	21.55 (18.36, 24.68)	22.29 (18.75, 24.43)	0.65
Laboratory findings					
Leucocyte (10^9/L)	6.11 (4.71, 8.61)	6.57 (4.88, 9.51)	7.41 (5.58, 11.24)	5.34 (3.79, 6·64)	0.003
Hemoglobin (g/L)	108.00 (90.50, 122.50)	119.00 (108.50, 131.50)	99.50 (88.25, 113.75)	95.00 (82.00, 108.00)	<0.001
Platelet (10^9/L)	165.00 (124.00, 214.00)	174.00 (135.00, 221.50)	148.50 (127.25, 254.50)	154.00 (111.50, 202.00)	0.145
Albumin (g/L)	35.30 (30.95, 39.00)	36.95 (33.27, 39.20)	31.55 (27.88, 34.70)	36.20 (33.10, 39.30)	<0.001
Alanine transaminase (U/L)	15.00 (8.00, 26.00)	18.00 (11.00, 28.00)	13.50 (9.00, 31.00)	10.00 (6.00, 20.25)	0.003
Aspartate transaminase (U/L)	24.00 (17.00, 34.00)	26.00 (22.00, 35.50)	27.00 (19.75, 48.00)	16.50 (13.00, 23.25)	<0.001
Total bilirubin (μmol/L)	10.10 (7.90, 14.25)	12.00 (8.85, 15.15)	10.35 (7.50, 15.40)	8.60 (7.40, 11.10)	<0.001
Creatinine (μmol/L)	131.00 (104.00, 187.00)	114.00 (99.50, 137.00)	258.50 (194.00, 382.50)	NA	<0.001
Fasting blood glucose (mmol/L)	6.72 (5.37, 9.19)	6.08 (5.25, 8.55)	7.34 (5.34, 9.05)	7.38 (5.54, 9.48)	0.03
C-reactive protein (mg/l)	16.13 (4.07, 46.57)	11.66 (3.00, 39.66)	27.45 (13.70, 84.74)	12.16 (3.59, 41.24)	0.178
Symptoms, n (%)					
Fever	26 (13.3)	10 (10.8)	7 (17.5)	9 (14.5)	0.55
Cough	39 (20.0)	8 (8.6)	13 (32.5)	18 (29.0)	<0.001
Sore throat	9 (4.6)	2 (2.2)	1 (2.5)	6 (9.7)	0.07
Stuffy running nose	5 (2.6)	0 (0)	2 (5.0)	3 (4.8)	0.096
Other	10 (5.1)	5 (5.4)	3 (7.5)	2 (3.2)	0.63
Clinical presentation^a^, n (%)					0.061
Mild/Moderate	190 (97.4)	91 (97.8)	37 (92.5)	62 (100.0)	
Severe/Critical	5 (2.6)	2 (2.2)	3 (7.5)	0	
Nirmatrelvir/ritonavir therapy, n (%)	73 (37.4)	45 (48.4)	9 (22.5)	19 (30.6)	0.007
Comorbidities, n (%)					
Hypertension	142 (72.8)	66 (71.0)	26 (65.0)	50 (80.6)	0.19
Diabetes Mellitus	62 (31.8)	26 (28.0)	12 (30.0)	24 (38.7)	0.36
Cardiovascular disease	110 (56.4)	59 (63.4)	29 (72.5)	22 (35.5)	<0.001
Chronic pulmonary disease	31 (15.9)	20 (21.5)	5 (12.5)	6 (9.7)	0.12
Chronic liver disease	8 (4.1)	5 (5.4)	3 (7·5)	0 0)	0·12
Malignancy	18 (9.2)	7 (7.5)	8 (20.0)	3 (4·8)	0·026
Other	36 (18.5)	18 (19.4)	15 (37.5)	3 (4.8)	<0.001
Comorbidity number>2, n (%)	132 (67.7)	65 (69.9)	35 (87.5)	32 (51.6)	<0.001
Medications, n (%)					
Renin angiotensin aldosterone system inhibitor	56 (28.7)	31 (33.3)	5 (12.5)	20 (32.3)	0.039
Calcium channel blocker	90 (46.2)	36 (38.7)	15 (37.5)	39 (62.9)	0.006
β-Blocker	49 (25.1)	19 (20.4)	6 (15.0)	24 (38.7)	0.009
Other antihypertensive agents	31 (15.9)	6 (6.5)	6 (15.0)	19 (30.6)	<0.001
Hypoglycemics	44 (22.6)	16 (17.2)	8 (20.0)	20 (32.3)	0.082
Lipid-lowering agents	45 (23.1)	18 (19.4)	10 (25.0)	17 (27.4)	0.48
Antiplatelet or anticoagulation agents	68 (34.9)	35 (37.6)	13 (32.5)	20 (32.3)	0.74
Immunosuppressants	6 (3.1)	3 (3.2)	1 (2.5)	2 (3.2)	0.97

eGFR, estimated glomerular filtration rate; NA, not applicable.

^a^ Clinical presentation is defined by the National guideline^22^.

### 3.2 Associations of nirmatrelvir/ritonavir uptake and outcomes

**FIGURE 1:**
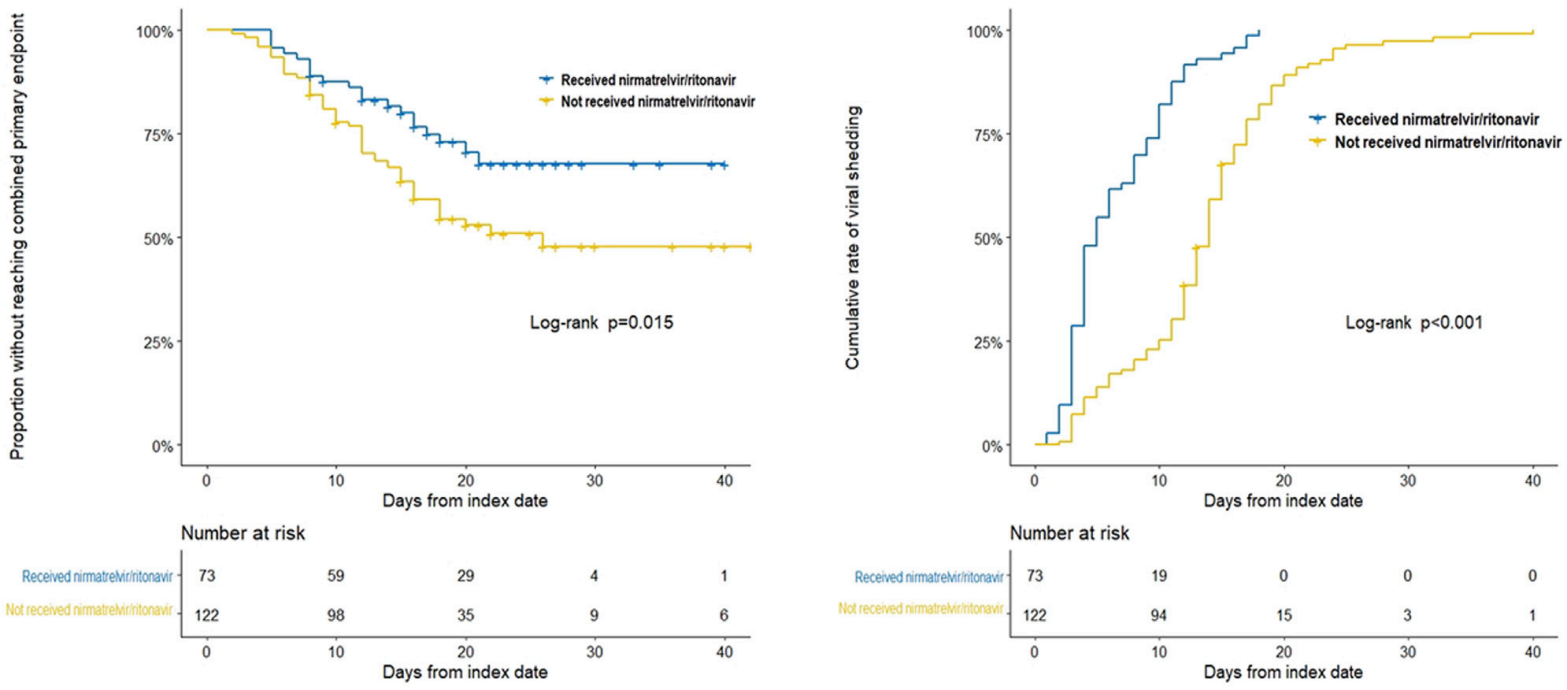
Kaplan-Meier analyses of time to the combined primary endpoint and the secondary endpoint in patients with SARS-CoV-2 infection and impaired kidney function with or without nirmatrelvir/ritonavir therapy. The Kaplan-Meier estimate of the duration from the respective index date to the combined primary endpoint and the secondary endpoint. The index date corresponded to the day of nirmatrelvir/ritonavir initiation for patients who received nirmatrelvir/ritonavir therapy, while for patients who did not receive nirmatrelvir/ritonavir, index date corresponded to the day of diagnosis of SARS-CoV-2 infection.

**FIGURE 2:**
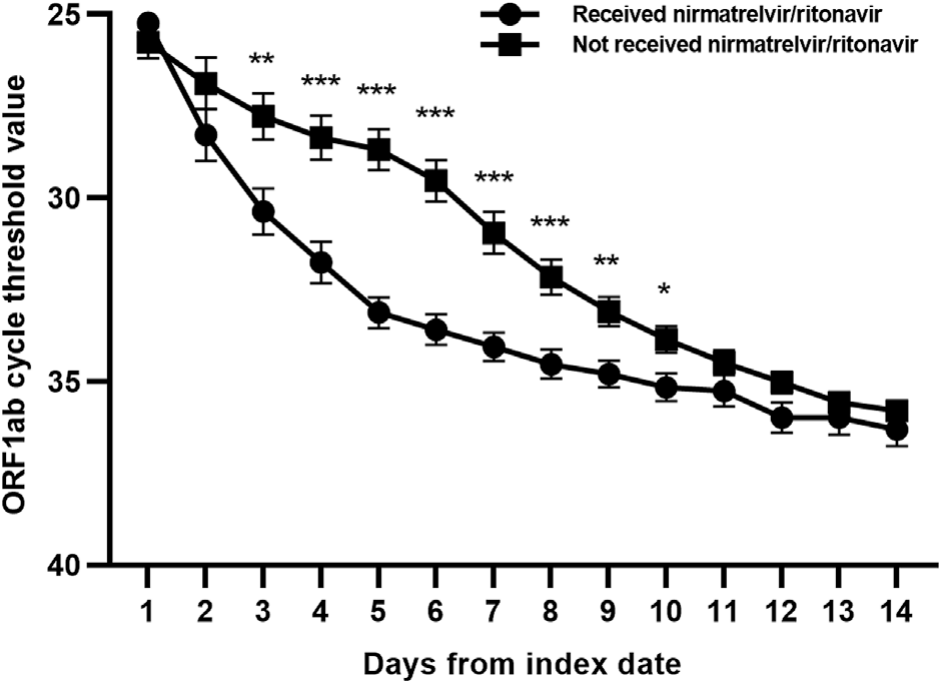
Changes of ORF1ab gene cycle threshold values in patients with SARS-CoV-2 infection and impaired kidney function with or without nirmatrelvir/ritonavir therapy. Data are mean (standard error of mean). ***p < 0.001; **p < 0.01; *p < 0.05.

We compared the clinical characteristics between the nirmatrelvir/ritonavir recipients and non-recipients (Table 2). No significant differences were found between the two groups concerning age, sex, and most of the baseline laboratory results. All the patients who received nirmatrelvir/ritonavir therapy had more comorbidities than those who did not receive nirmatrelvir/ritonavir. Medication use was generally comparable between the two groups, except for higher rate of use of renin angiotensin aldosterone system inhibitor (RAASi), lipid-lowering agents, and antiplatelet or anticoagulation agents in nirmatrelvir/ritonavir recipients. The proportion of patients who reached the combined primary endpoint of all-cause mortality, ICU admission, or cardiovascular events was lower in nirmatrelvir/ritonavir recipients than in controls [20 (27.4%) versus 57 (46.7%), *p* = 0.008] (Table 2). A lower risk of the combined primary endpoint was observed in nirmatrelvir/ritonavir recipients compared with non-nirmatrelvir/ritonavir recipients [adjusted HR 0.56 (95% CI: 0.33–0.95); *p =* 0.032] (Table 3). Patients who received nirmatrelvir/ritonavir therapy experienced a shorter duration of viral shedding [5.00 days [3.00, 10.00] vs. 13.00 days (10.00, 17.00), *p* < 0.001], shorter length of hospital stay [16.00 days (11.00, 22.00) vs. 17.50 days [14.00, 23.00], *p* = 0·026], and faster viral load clearance versus patients who did not receive nirmatrelvir/ritonavir (Table 2; Figures 1, 2). Further, Cox regression analysis showed that nirmatrelvir/ritonavir therapy was associated with shorter viral shedding time [adjusted HR 4.57 (95% CI: 2.86–7.30); *p* < 0·001] (Table 3).

**TABLE 2:** Characteristics of patients with SARS-CoV-2 infection and impaired kidney function with or without Nirmatrelvir/ritonavir therapy.

	Received nirmatrelvir/ritonavir (*n* = 73)	Not received nirmatrelvir/ritonavir (*n* = 122)	*p*
Age (year)	77.00 (64.00, 87.00)	73.00 (64.00, 83.00)	0.091
Sex male, n (%)	41 (56.2)	68 (55.7)	0.95
BMI (kg/m^2^)	21.55 (18.78, 24.39)	21.22 (18.75, 24.17)	0.66
Laboratory findings			
Leucocyte (10^9/L)	6.08 (4.29, 8.89)	6.10 (4.85, 8.14)	0.81
Hemoglobin (g/L)	111.00 (95.00, 127.00)	106.00 (85.00, 119.00)	0.036
Platelet (10^9/L)	154.00 (120.50, 191.50)	166.00 (124.00, 213.00)	0.91
Albumin (g/L)	36.20 (31.95, 39.05)	35.10 (30.60, 39.20)	0.92
Alanine transaminase (U/L)	17.00 (11.00, 29.50)	12.50 (7.00, 24.00)	0.01
Aspartate transaminase (U/L)	26.00 (18.00, 38.00)	23.00 (16.00, 33.00)	0.15
Total bilirubin (μmol/L)	9.80 (7.95, 13.60)	10.25 (7.90, 14.70)	0.74
Fasting blood glucose (mmol/L)	7.28 (5.56, 9.13)	6.63 (5.32, 9.39)	0.34
C-reactive protein (mg/l)	12.60 (4.36, 46.58)	19.38 (4.25, 41.62)	0.70
Baseline ORF1ab cycle threshold value^a^	25.23 ± 3.20	25.76 ± 4.76	0.35
Baseline N cycle threshold value^a^	25.16 ± 3.36	25.82 ± 4·82	0.31
Creatinine (μmol/L) (*n* = 133)^b^	122.00 (102.50, 155.50)	147.00 (107.00, 211.50)	0.016
eGFR (ml/min/1·73 m^2^) (*n* = 133) ^b^	46.00 (34.00, 55.00)	36.00 (20.50, 47.50)	0.003
eGFR category			0.007
30≤eGFR<60 (ml/min/1·73 m^2^)	45 (61.6)	48 (39.3)	
eGFR<30 (ml/min/1·73 m^2^) (Non-dialysis)	9 (12.3)	31 (25.4)	
Maintenance hemodialysis	19 (26.0)	43 (35.2)	
Clinical presentation, n (%)			0.41
Mild/Moderate	72 (98.6)	118 (96.7)	
Severe/Critical	1 (1.4)	4 (3.3)	
Comorbidities, n (%)			
Hypertension	57 (78.1)	85 (69.7)	0.20
Diabetes Mellitus	27 (37.0)	35 (28.7)	0.23
Cardiovascular disease	47 (64.4)	63 (51.6)	0.082
Chronic pulmonary disease	15 (20.5)	16 (13.1)	0.17
Chronic liver disease	0 (0)	8 (6.6)	0.025
Malignancy	5 (6.8)	13 (10.7)	0.37
Other	15 (20.5)	21 (17.2)	0.56
Comorbidity number>2, n (%)	57 (78.1)	75 (61.5)	0.016
Medications, n (%)			
Renin angiotensin aldosterone system inhibitor	29 (39.7)	27 (22.1)	0.014
Calcium channel blocker	34 (46.6)	56 (45.9)	0.52
β-Blocker	21 (28.8)	28 (23.0)	0.23
Other antihypertensive agents	13 (17.8)	18 (14.8)	0.36
Hypoglycemics	20 (27.4)	24 (19.7)	0.14
Lipid-lowering agents	24 (32.9)	21 (17.2)	0.014
Antiplatelet or anticoagulation agents	37 (50.7)	31 (25.4)	<0.001
Immunosuppressants	4 (5.5)	2 (1.6)	0.20
Outcomes			
Combined primary endpoint^a^	20 (27.4)	57 (46.7)	0.008
All-cause death, n (%)	7 (9.6)	17 (13.9)	0.37
ICU admission, n (%)	17 (23.3)	33 (27.0)	0.56
Cardiovascular events, n (%)	13 (17.8)	41 (33.6)	0.017
Duration of viral shedding (day)^c^	5.00 (3.00, 10.00)	13.00 (10.00, 17.00)	<0.001
Length of hospital stay (day)	16.00 (11.00, 22.00)	17.50 (14.00, 23.00)	0.026

eGFR, estimated glomerular filtration rate.

^a^ The index date corresponded to the day of Nirmatrelvir/ritonavir initiation for patients who received Nirmatrelvir/ritonavir therapy, while for patients who did not receive Nirmatrelvir/ritonavir, index date corresponded to the day of diagnosis of SARS-CoV-2, infection. The baseline levels of both ORF1ab and N genes cycle threshold values were not statistical significant between the two groups.

^b^ Maintenance hemodialysis patients were not included.

^c^ The duration of viral shedding was defined as the time interval from the respective index date to the day of viral shedding.

**TABLE 3:** Outcomes for patients with SARS-CoV-2 infection and impaired kidney function with and without Nirmatrelvir/ritonavir therapy.

	Unadjusted HR (95% CI)	Model 1^a^ HR (95% CI)	Model 2^b^ HR (95% CI)
Combined primary endpoint	0.54 (0.33–0.90)	0.51 (0.30–0.85)	0.56 (0.32–0.96)
*p*	0.018	0.01	0.035
Viral shedding	5.16 (3.32–8.01)	5.29 (3.39–8.25)	3.70 (2.60–5.28)
*p*	<0.001	<0.001	<0.001

The association between Nirmatrelvir/ritonavir therapy and the outcomes was estimated by multivariate Cox proportional-hazards regression model after adjustment for confounders. The variables were age, sex, and eGFR, category, and comorbidity number.

^a^ Model 1 was adjusted for age and sex.

^b^ Model 2 was adjusted for Model 1 plus eGFR, category, and comorbidity number.

HR, hazard ratio; CI, confidence interval; eGFR, estimated glomerular filtration rate.

### 3.3 Associations of the timing of nirmatrelvir/ritonavir initiation and outcomes

We divided the nirmatrelvir/ritonavir recipients according to the timing of nirmatrelvir/ritonavir initiation. No significant difference was observed in the proportion of patients who reached the combined primary endpoint in nirmatrelvir/ritonavir recipients and controls [9 (25.0%) versus 11 (29·7%), *p* = 0.65] (Supplementary Table S1). Nirmatrelvir/ritonavir prescription within 5 days of diagnosis resulted in a shorter duration of viral shedding [8.50 days (5.50, 11.50) vs. 17.00 days (13.00, 22.00), *p* < 0.001], higher viral shedding rate within 10 and 14 days (69.4% vs. 13.5%, *p* < 0.001 and 94.4% vs. 32.4%, *p* < 0.001, respectively), shorter length of hospital stay [14.00 days (10.00, 20.50) vs. 19.00 days (15.00, 26.00), *p* = 0.003], and faster viral load clearance (Supplementary Table S1; Supplementary Figures S2–S4). Cox regression analysis showed that earlier initiation of nirmatrelvir/ritonavir within 5 days since the diagnosis of SARS-CoV-2 infection was associated with shorter viral shedding time compared to late initiation [adjusted HR 7.84 (95% CI: 3.28–18.76); *p* < 0.001] (Supplementary Table S2). In addition, the timing of nirmatrelvir/ritonavir initiation and length of hospital stay had a linear correlation (*p* < 0.001) (Supplementary Figure S5).

### 3.4 Safety information

We compared the leucocyte, platelet, alanine transaminase, aspartate transaminase, total bilirubin, and eGFR levels at baseline and 10 days after nirmatrelvir/ritonavir initiation (Supplementary Figure S6). A total of five (6.8%) adverse events were reported among the nirmatrelvir/ritonavir recipients, including one mild liver dysfunction with elevated alanine transaminase and aspartate transaminase, one acute kidney injury, two patients experiencing vomiting, and one patient having transient dysgeusia. All of them were resolved without further intervention. Significant difference was seen between the platelet levels at baseline and 10 days after nirmatrelvir/ritonavir initiation, while they were all stable or varying within the normal range. Seven patients died during hospitalization. The time of death was all beyond 14 days after the end of the nirmatrelvir/ritonavir course. Medical group’s discussions on death cases considered that the deaths were most probably due to the progression of their underlying comorbidities (one diabetic ketosis, one cerebral hemorrhage, one malignancy dyscrasia, two acute on chronic heart failure, and two severe bacteria pneumonia cases). Overall, no patient reported serious adverse events that called for the suspension of nirmatrelvir/ritonavir therapy.

## 4 Discussion

We have shown that treatment with nirmatrelvir/ritonavir for high-risk patients with impaired kidney function was related with a lower risk of the composite of all-cause mortality, ICU admission, or cardiovascular events, as well as markedly reduced duration of viral shedding, a quick reduction in viral load, and a shorter length of hospital stay. Earlier initiation of nirmatrelvir/ritonavir within 5 days of SARS-CoV-2 infection diagnosis yielded further efficacy in reducing viral shedding time compared with late initiation. No serious adverse events were reported among nirmatrelvir/ritonavir recipients.

It was worth noting that this investigation was carried out in Shanghai, China while Omicron BA.2.2 was the dominant variant and demonstrated the efficacy of nirmatrelvir/ritonavir in treating the infection by Omicron sub-lineage. Previous data demonstrated that Omicron variant tended to result in more infections while less serious cases. However, repeated outbreaks of the pandemic and the large population base of the vulnerable individuals brought the medical system overwhelmed (Carlson et al., 2021; Zhang et al., 2022a; Gao et al., 2022; Lu et al., 2022). Preventing poor outcomes and decreasing the course of the disease with nirmatrelvir/ritonavir therapy could be an optimal option to fight against COVID-19.

Both indication and intervention time are key factors for antiviral medication usage. To date, nirmatrelvir/ritonavir is recommended for non-hospitalized patients with a high risk of progressing to serious COVID-19 cases within 5 days since the occurence of symptoms and yielded satisfactory effectiveness. However, our real-world study focuses on its efficacy in hospitalized patients. Because under the local prescription medication regulation and city-wide management and control, patients did not have access to nirmatrelvir/ritonavir until they were transferred to a COVID-19 referral hospital. Thus a proportion of patients received nirmatrelvir/ritonavir beyond 5 days since the established diagnosis of SARS-CoV-2 infection. It should be noted that nirmatrelvir/ritonavir might be prescribed earlier in the EPIC-HR study than our real-world cohort. Nirmatrelvir/ritonavir was administered within 5 days since symptom onset in the EPIC-HR trial, whereas in our cohort, participants were enrolled up to 5 days since the laboratory confirmation of SARS-CoV-2 infection.

In comparison between the nirmatrelvir/ritonavir users and non-users, our study demonstrated the association of nirmatrelvir/ritonavir therapy with a lower risk of the composite of all-cause mortality, ICU admission, or cardiovascular events, and also a shortened duration of SARS-CoV-2 viral shedding in patients with impaired kidney function. The results are in consistent with the previous studies which has revealed positive role of nirmatrelvir/ritonavir therapy in treating patients with SARS-CoV-2 infection (Mahase, 2021; Owen et al., 2021; Arbel et al., 2022; Hammond et al., 2022). However, studies focusing on patients with impaired kidney function, particularly those with an eGFR <45 ml/min/1·73 m^2^ and maintenance hemodialysis patients, are still limited and covering small sample size (Lingscheid et al., 2022; Toussi et al., 2022). Previous studies reported the relationship of CKD with poor outcomes in COVID-19 (Ozturk et al., 2020; Carlson et al., 2021; Flythe et al., 2021; Russo et al., 2021). Notably, we included eGFR as a confounding variable in cox regression analysis, which suggested that the effectiveness of nirmatrelvir/ritonavir was regardless of the severity of kidney dysfunction.

Owing to the pandemic prevention and control measures and the prescription medication regulation of the government, 37 (50.7%) of the nirmatrelvir/ritonavir recipients in our cohort initiated the drug administration beyond 5 days since the diagnosis of SARS-CoV-2 infection. Moreover, These patients started nirmatrelvir/ritonavir intervention at a median 11.0 days since the diagnosis. The time interval from the diagnosis of SARS-CoV-2 infection to the initiation of nirmatrelvir/ritonavir therapy can affect the duration of viral elimination. Thus, we did further analysis to examine the associations of the timing of nirmatrelvir/ritonavir initiation and the patient outcomes. Our study demonstrated the association of earlier initiation of nirmatrelvir/ritonavir therapy within 5 days of SARS-CoV-2 infection diagnosis with a shortened viral shedding time compared with late nirmatrelvir/ritonavir initiation, regardless of the severity of kidney dysfunction. However, we did not find significant association of earlier initiation of nirmatrelvir/ritonavir with the combined primary outcomes, probably due to the retrospective study in nature and the small sample size.

Israel was among the first countries in the world to apply large-scale administration of new oral antiviral medications. Data revealed few and mild side effects resulted from the drug (Arbel et al., 2022). Among the reported adverse events, the most common ones were diarrhea and dysgeusia. Other adverse events included liver dysfunction, abnormal D-dimer levels, nausea, and headache, most of which were grade 1 or 2 and could resolve spontaneously in a short time. Few patients experienced serious adverse events, such as increased serum creatinine level and COVID-19 pneumonia. To date, particularly in patients with underlying comorbidities, safety information of the new oral antivirals in the clinical setting are still limited. The concomitant administration of nirmatrelvir/ritonavir and certain medications might cause potentially harmful drug interaction and increase the risk of adverse reactions (Fact Sheet for Healthcare Providers, 2022). For the safety concern, we consulted the pharmacist about the prescription and dose adjustment of nirmatrelvir/ritonavir in patients with severely impaired kidney function. In our cohort, five (6.8%) patients experienced mild grade adverse events. Seven patients died beyond 14 days after the end of the nirmatrelvir/ritonavir course and most probably due to the progression of their underlying comorbidities by the attending physician’s clinical judgement. It is worth noting that the mortality rate of the nirmatrelvir/ritonavir group was lower than the non-nirmatrelvir/ritonavir group (9.6% vs. 13.9%). Concerning the medication use in the cohort, we noticed a higher rate of usage of RAASi, lipid-lowering agents, and antiplatelet or anticoagulation agents in nirmatrelvir/ritonavir recipients. It is reported that angiotensin converting enzyme 2 serves as the target receptor of SARS-CoV-2 and RAASi may be associated with reduced mortality in COVID-19, however there are no consistent conclusions among different studies (Gnanenthiran et al., 2022; Wang et al., 2022). The patients who used lipid-lowering agents, and antiplatelet or anticoagulation agents were those who had concomitant hypertension, diabetes or cardiovascular disease. The patients took these drugs regularly and the medication use suggested the burden of comorbidities. Therefore, we included comorbidity number in the multivariate analysis to address the confounding effect of underlying diseases.

Our study is a preliminary investigation of the efficacy and safety of nirmatrelvir/ritonavir therapy in patients with impaired kidney function in the real world, aiming to provide evidence in this field. Recent data showed promising pharmaceutical application of nano materials in the management of COVID-19, including early detection of the infection and targeted delivery of antivirals (Nahhas and Webster, 2021; Zhu et al., 2021). We may expect that the new technique would play a role in enhancing antiviral drug efficacy and decreasing adverse reactions. Our study has several limitations. 1) This was a single center study. The results in our cohort only represent the specific patient population and do not extend to all the patients infected with Omicron in the world. 2) We applied viral shedding duration in the assessment of the efficacy of nirmatrelvir/ritonavir therapy, while not every patient received the diagnosis on the first day of infection. 3) As this was a retrospective study, various confounding variables might create bias in the results. We have attempted to adjust several important variables including comorbidities, yet we could not exclude all potential interference factors. 4) The safety analysis of nirmatrelvir/ritonavir therapy in our study was preliminary. Owing to the retrospective and non-controlled design, we were not able to analyze the safety information concisely. 5) It is necessary to design randomized controlled trial and prospective experiment and establish long-term observation to clarify the exact effectiveness, pharmacokinetics, and safety of nirmatrelvir/ritonavir application in patients with severely impaired kidney function. 6) Our sample size was small. Further investigations are needed to examine larger groups of patients.

## 5 Conclusion

In summary, our findings supported the early initiation of nirmatrelvir/ritonavir therapy for high-risk patients with impaired kidney function. This could facilitate improved patient outcomes, faster viral shedding, shorter length of hospital stay, and reduced need for medical resources. No evident safety concerns were noted in our cohort during hospitalization. However, large and long-term follow-up data are needed for verification.

## Data Availability

The raw data supporting the conclusion of this article will be made available by the authors, without undue reservation.
