# Supplementary material for: Nirmatrelvir/ritonavir for patients with SARS-CoV-2 infection and impaired kidney function during the Omicron surge
Source: Front Pharmacol. 2023 Mar 22;14:1147980. doi: 10.3389/fphar.2023.1147980 (PMC10073454; doi:10.3389/fphar.2023.1147980)

**Supplementary appendix**

**Nirmatrelvir/ritonavir for patients with SARS-CoV-2 infection and impaired kidney function during the Omicron surge**

**Table of Contents**

Figure S1. Assessment for eligibility………………………………………………………………………….2

Table S1. Characteristics of patients with SARS-CoV-2 infection and impaired kidney function prescribed with nirmatrelvir/ritonavir within or beyond five days since the diagnosis of SARS-CoV-2 infection………3

Figure S2. Cumulative rate of viral shedding in patients with SARS-CoV-2 infection and impaired kidney function prescribed with nirmatrelvir/ritonavir within or beyond five days since the diagnosis of SARS-CoV-2 infection…………………………………………………………………………………………………..…5

Figure S3. Viral shedding rate of patients with SARS-CoV-2 infection and impaired kidney function prescribed with nirmatrelvir/ritonavir within or beyond five days since the diagnosis of SARS-CoV-2 infection……………….………………………………………………………………………………………6

Figure S4. Changes of ORF1ab gene cycle threshold values in patients with SARS-CoV-2 infection and impaired kidney function prescribed with nirmatrelvir/ritonavir within or beyond five days since the diagnosis of SARS-CoV-2 infection…………………………………………………………………………………….7

Table S2. COX regression analysis of the correlation between the timing to initiate nirmatrelvir/ritonavir and viral shedding in patients with SARS-CoV-2 infection and impaired kidney function………………………8

Figure S5. Correlation between the timing to initiate nirmatrelvir/ritonavir and length of hospital stay in patients with SARS-CoV-2 infection and impaired kidney function…………………………………………9

Figure S6. Safety of nirmatrelvir/ritonavir therapy in patients with SARS-CoV-2 infection and impaired kidney function……………………………………………………………………………………………….10

**Figure S1. Assessment for eligibility.**


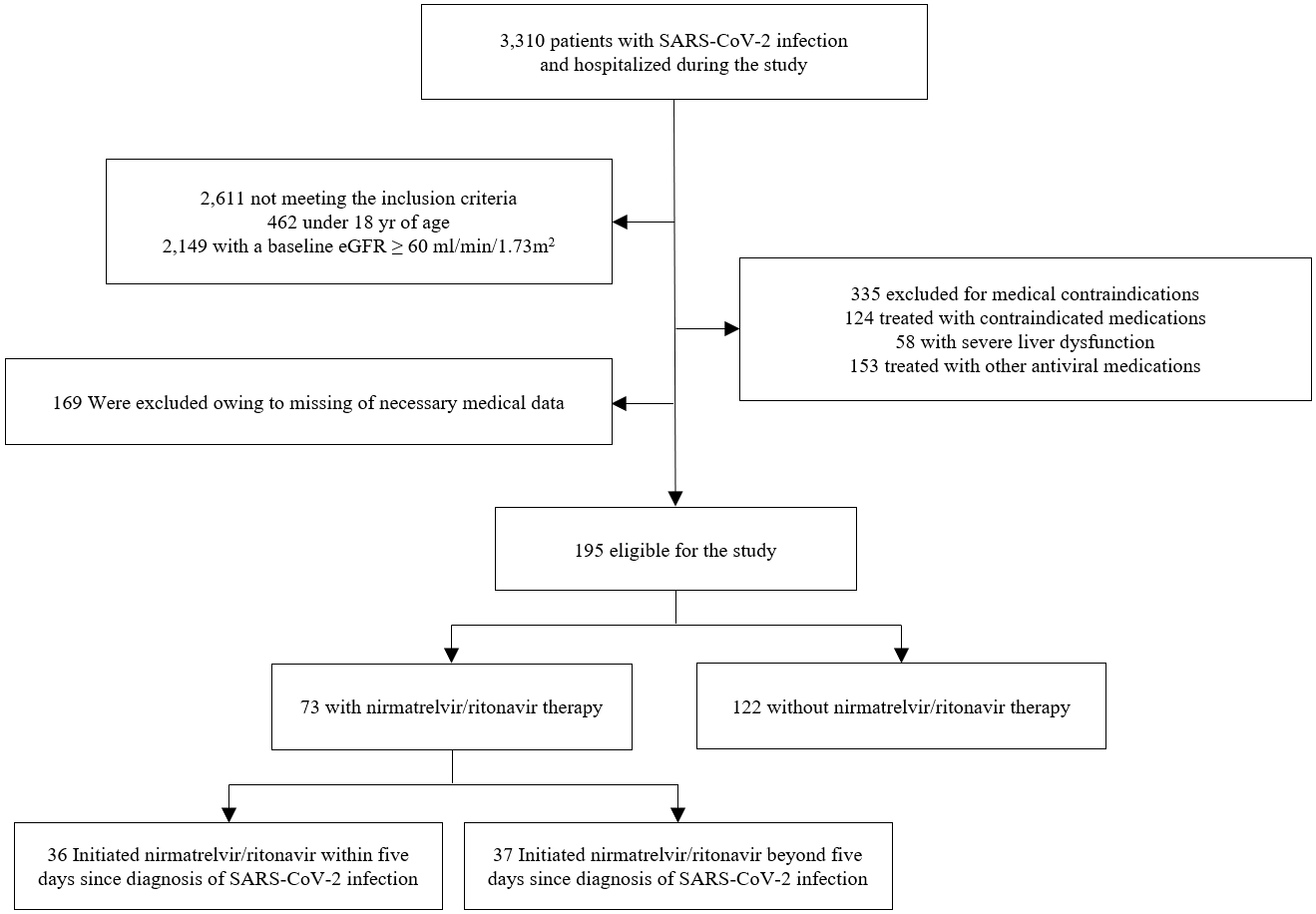


SARS-CoV-2: severe acute respiratory syndrome coronavirus 2; eGFR: Estimated glomerular filtration rate

**Table S1. Characteristics of patients with SARS-CoV-2 infection and impaired kidney function prescribed with nirmatrelvir/ritonavir within or beyond five days since the diagnosis of SARS-CoV-2 infection.**

|  | **Total**  **(n=73)** | **Initiation of nirmatrelvir/ritonavir≤5days**  **(n=36)** | **Initiation of nirmatrelvir/ritonavir>5days**  **(n=37)** | ***p*** |
| --- | --- | --- | --- | --- |
| **Age (year)** | 77·00 (64·00, 88·00) | 85·50 (65·00, 91·00) | 84·00 (77·50, 87·50) | 0·42 |
| **Sex Male, n (%)** | 41 (56·2) | 21 (58·3) | 20 (54·1) | 0·71 |
| **BMI (kg/m^2^)** | 21·55 (18·78, 24·39) | 21·08 (18·95, 24·43) | 22·32 (18·47, 24·33) | 0.74 |
| **Laboratory findings** |  |  |  |  |
| **Leucocyte (10^9/L)** | 6·36 (4·19, 10·03) | 5·91 (4·19, 8·61) | 7·66 (6·26, 12·65) | 0·57 |
| **Hemoglobin (g/L)** | 113·00 (95·00, 126·00) | 125·00 (109·00, 132·00) | 118·00 (106·00, 125·50) | 0·01 |
| **Platelet (10^9/L)** | 162·00 (131·00, 207·00) | 144·50 (123·00, 178·00) | 162·00 (142·50, 187·50) | 0·85 |
| **Albumin (g/L)** | 36·20 (30·57, 38·93) | 37·10 (33·20, 39·10) | 32·70 (28·15, 39·10) | 0·61 |
| **Alanine transaminase (U/L)** | 19·00 (11·00, 28·00) | 18·50 (10·00, 28·00) | 21·00 (15·00, 55·50) | 0·91 |
| **Aspartate transaminase (U/L)** | 26·00 (18·00, 36·00) | 28·50 (23·0, 41·0) | 31·00 (19·00, 48·50) | 0·23 |
| **Total bilirubin (μmol/L)** | 9·80 (7·90, 13·70) | 11·70 (8·55, 13·85) | 9·00 (7·70, 12·20) | 0·19 |
| **Fasting blood glucose (mmol/L)** | 7·23 (5·53, 9·13) | 6·21 (5·21, 9·09) | 7·33 (5·71, 9·09) | 0·41 |
| **C-reactive protein (mg/l)** | 13·88 (4·32, 57·00) | 15·01 (4·76, 32·46) | 28·60 (7·38, 62·83) | 0·67 |
| **Baseline ORF1ab cycle threshold value ^a^** | 24·90±5·57 | 24·35±5·60 | 25·44±5·56 | 0·41 |
| **Baseline N cycle threshold value ^a^** | 24·57±5·43 | 24·01±5·56 | 25·12±5·32 | 0·38 |
| **Creatinine (****μmol/L) (n=54) ^b^** | 122·00 (102·25, 157·75) | 117·00 (101·00, 160·00) | 124·00 (103·00, 151·00) | 0·68 |
| **eGFR (ml/min/1·73m^2^) (n=54) ^b^** | 46·00 (33·75, 55·00) | 41·00 (33·00, 55·00) | 48·00 (38·50, 56·00) | 0·85 |
| **eGFR category** |  |  |  | 0·067 |
| **30≤eGFR<60 (ml/min/1·73m^2^)** | 45 (61·6) | 26 (72·2) | 19 (51·4) |  |
| **eGFR<30 (ml/min/1·73m^2^) (Non-dialysis)** | 9 (12·3) | 5 (13·9) | 4 (10·8) |  |
| **Maintenance hemodialysis** | 19 (26·0) | 5 (13·9) | 14 (37·8) |  |
| **Comorbidities, n (%)** |  |  |  |  |
| **Hypertension** | 57 (78·1) | 29 (80·6) | 28 (75·7) | 0·61 |
| **Diabetes Mellitus** | 27 (37·0) | 11 (30·6) | 16 (43·2) | 0·26 |
| **Cardiovascular disease** | 47 (64·4) | 22 (61·1) | 25 (67·6) | 0·57 |
| **Chronic pulmonary disease** | 15 (20·5) | 9 (25·0) | 6 (16·2) | 0·35 |
| **Chronic liver disease** | 0 (0) | 0 (0) | 0 (0) |  |
| **Malignancy** | 5 (6·8) | 3 (8·3) | 2 (5·4) | 0·62 |
| **Other** | 15 (20·5) | 6 (16·7) | 9 (24·3) | 0·42 |
| **Comorbidity number>2, n (%)** | 57 (78·1) | 28 (77·8) | 29 (78·4) | 0·95 |
| **Outcomes ^a^** |  |  |  |  |
| **Duration of viral shedding (day)** | 12·00 (8·00, 17·50) | 8·50 (5·50, 11·50) | 17·00 (13·00, 22·00) | <0·001 |
| **Viral shedding within 10 days, n (%)** | 30 (41·1) | 25 (69·4) | 5 (13·5) | <0·001 |
| **Viral shedding within 14 days, n (%)** | 46 (63·0) | 34 (94·4) | 12 (32·4) | <0·001 |
| **Length of hospital stay (day)** | 17·00 (11·00, 24·00) | 14·00 (10·00, 20·50) | 19·00 (15·00, 26·00) | 0·003 |
| **Combined endpoint** | 20 (27·4) | 9 (25·0) | 11 (29·7) | 0·65 |
| **All-cause death, n (%)** | 7 (9·6) | 2 (5·6) | 5 (13·5) | 0·25 |
| **ICU admission, n (%)** | 17 (23·3) | 8 (22·2) | 9 (24·3) | 0·83 |
| **Cardiovascular events, n (%)** | 13 (17·8) | 6 (16·7) | 7 (18·9) | 0·80 |

eGFR: Estimated glomerular filtration rate

^a^ The index date of both group corresponded to the day of diagnosis of SARS-Cov-2 infection.

^b^ Maintenance hemodialysis patients were not included.

**Figure S2. Cumulative rate of viral shedding in patients with SARS-CoV-2 infection and impaired kidney function prescribed with nirmatrelvir/ritonavir within or beyond five days since the diagnosis of SARS-CoV-2 infection.**

The Kaplan-Meier estimate of the duration from the index date to the day of viral shedding. The index date of both group corresponded to the day of diagnosis of SARS-Cov-2 infection.


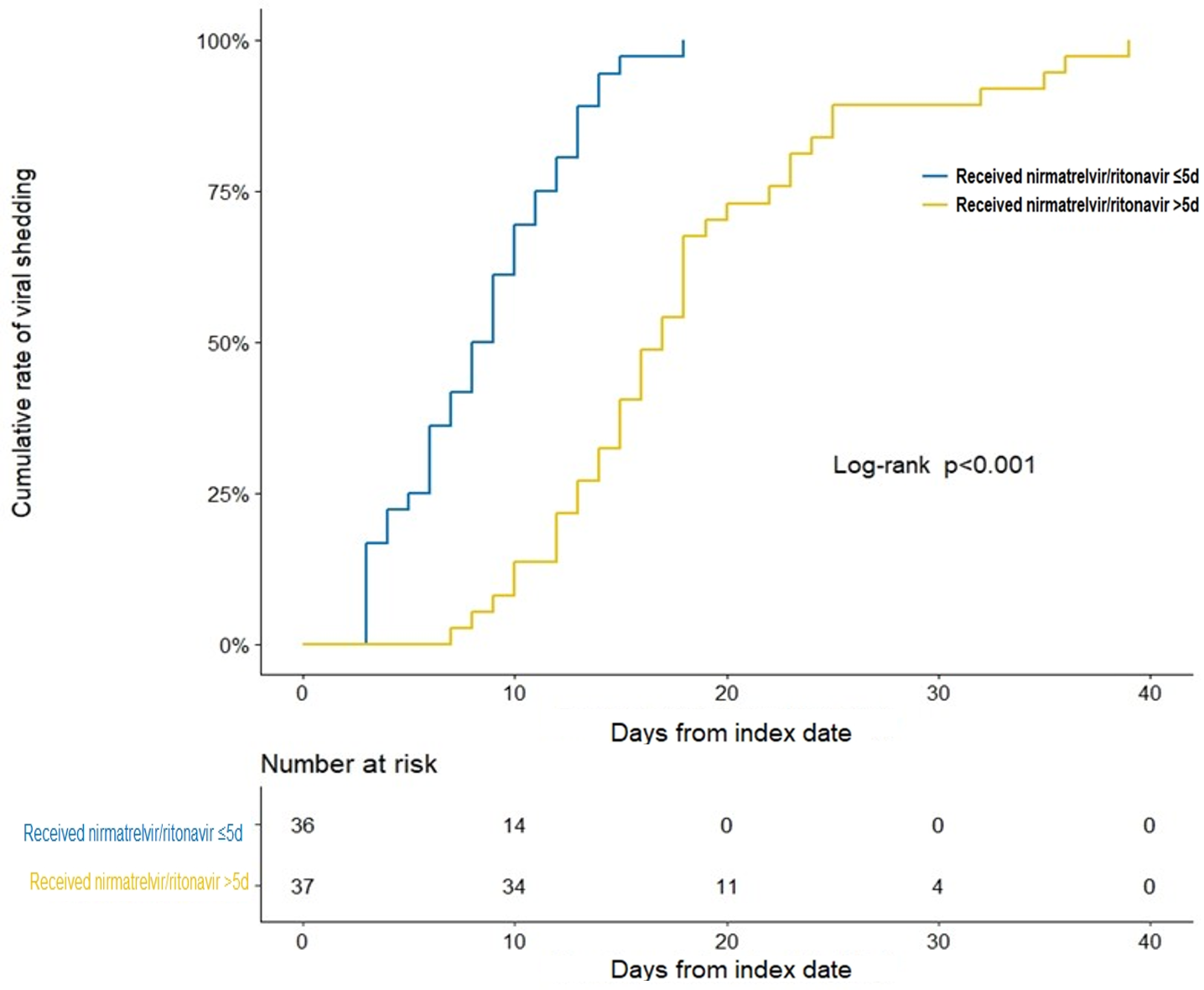


**Figure S3. Viral shedding rate of patients with SARS-CoV-2 infection and impaired kidney function prescribed with nirmatrelvir/ritonavir within or beyond five days since the diagnosis of SARS-CoV-2 infection.**


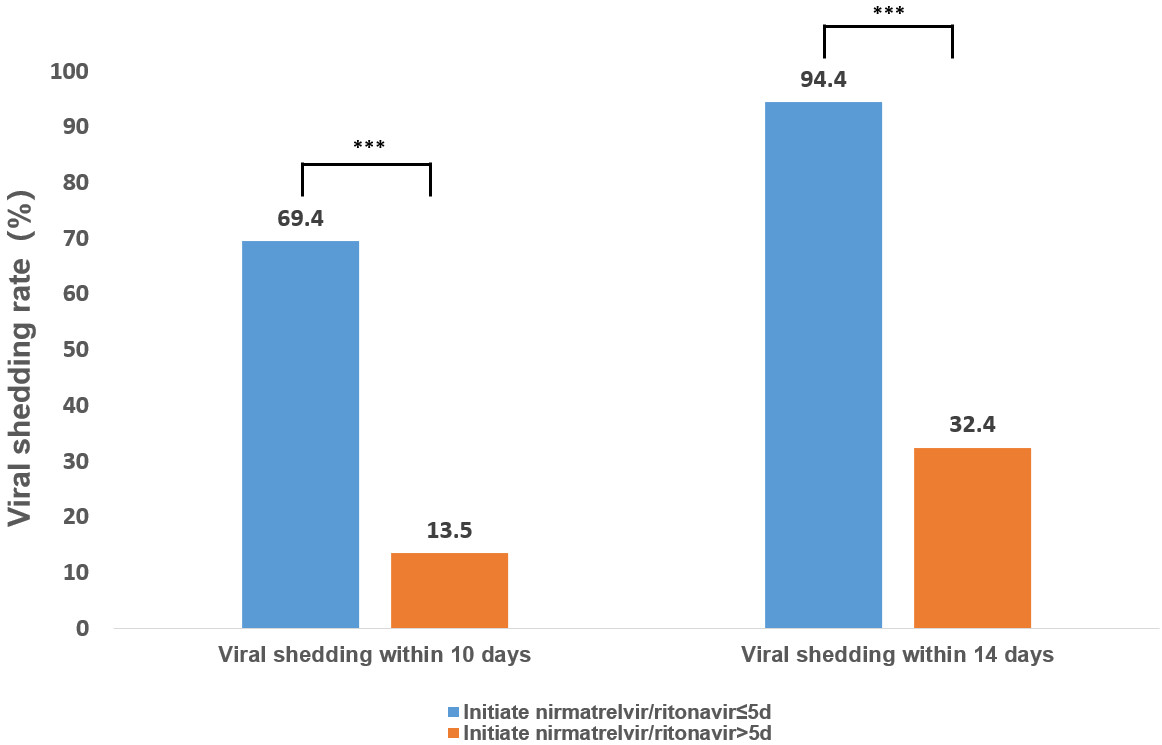


*** p<0·001

**Figure S4. Changes of ORF1ab gene cycle threshold values in patients with SARS-CoV-2 infection and impaired kidney function prescribed with nirmatrelvir/ritonavir within or beyond five days since the diagnosis of SARS-CoV-2 infection.**


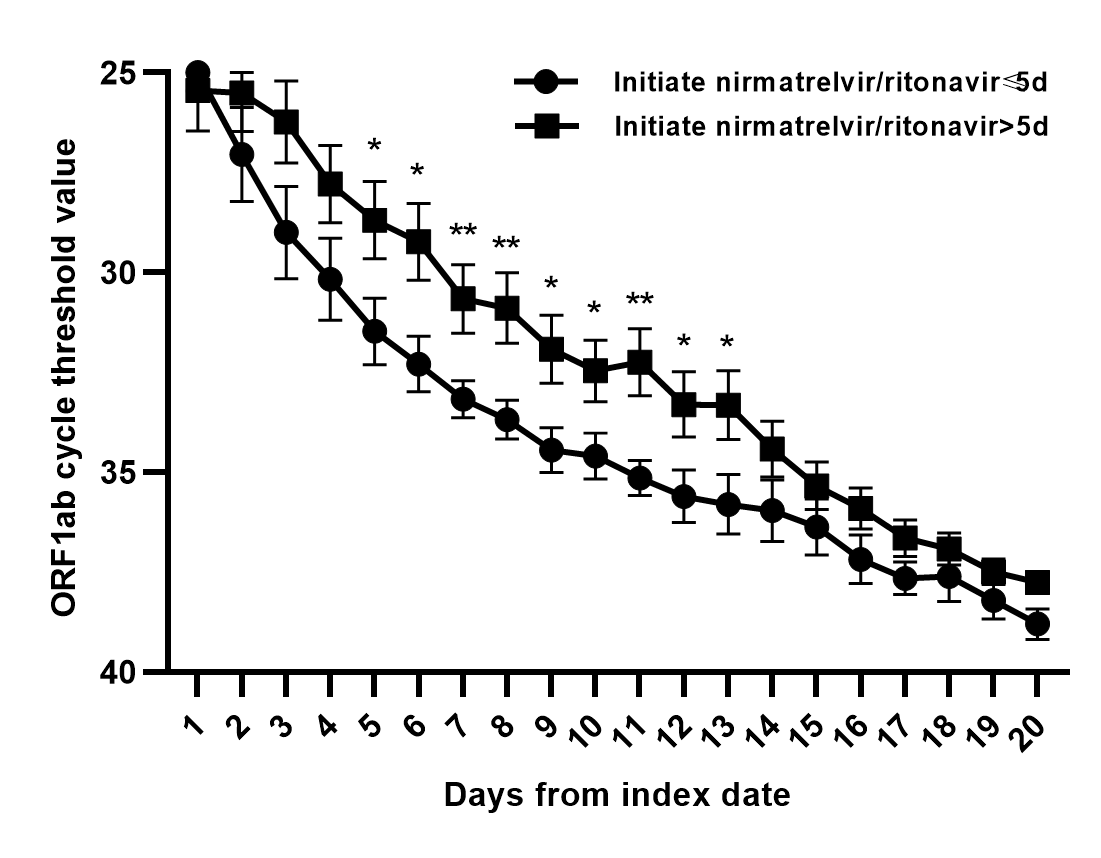


Data are mean (standard error of mean)

** p<0·01; * p<0·05

**Table S2. COX regression analysis of the correlation between the timing to initiate nirmatrelvir/ritonavir and viral shedding in patients with SARS-CoV-2 infection and impaired kidney function.**

|  | **Unadjusted HR (95% CI)** | **Model 1^a^ HR (95% CI)** | **Model 2^b^ HR (95% CI)** |
| --- | --- | --- | --- |
| **Initiation of nirmatrelvir/ritonavir>5days** | 1·00 (reference) | 1·00 (reference) | 1·00 (reference) |
| **Initiation of nirmatrelvir/ritonavir≤5days** | 6·86 (3·10~15·16) | 7·71 (3·36~17·70) | 7·84 (3·28~18·76) |
| ***p*** | <0·001 | <0·001 | <0·001 |

The association between the timing to initiate Nirmatrelvir/ritonavir and viral shedding was estimated by multivariate Cox proportional-hazards regression model after adjustment for confounders, including age, sex, and eGFR.

^a^ Model 1 was adjusted for age and sex

^b^ Model 2 was adjusted for Model 1 plus eGFR category

CI: confidence interval; eGFR: Estimated glomerular filtration rate

**Figure S5. Correlation between the timing to initiate nirmatrelvir/ritonavir and** **length of hospital stay in patients with SARS-CoV-2 infection and impaired kidney function.**


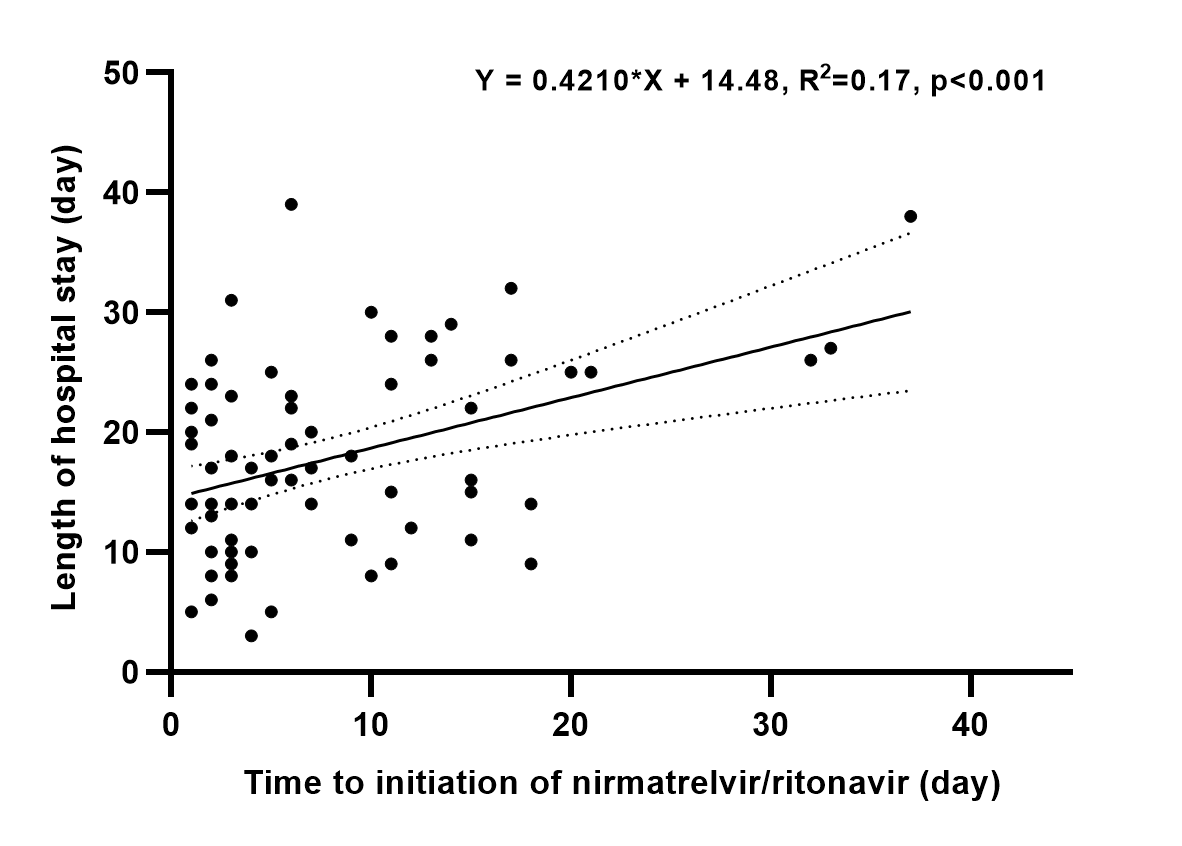


**Figure S6. Safety of nirmatrelvir/ritonavir therapy in patients with SARS-CoV-2 infection and impaired kidney function.**

Comparison of leucocyte, platelet, alanine transaminase, aspartate transaminase, total bilirubin, and estimated glomerular filtration rate (eGFR) levels at baseline and 10 days after nirmatrelvir/ritonavir initiation.


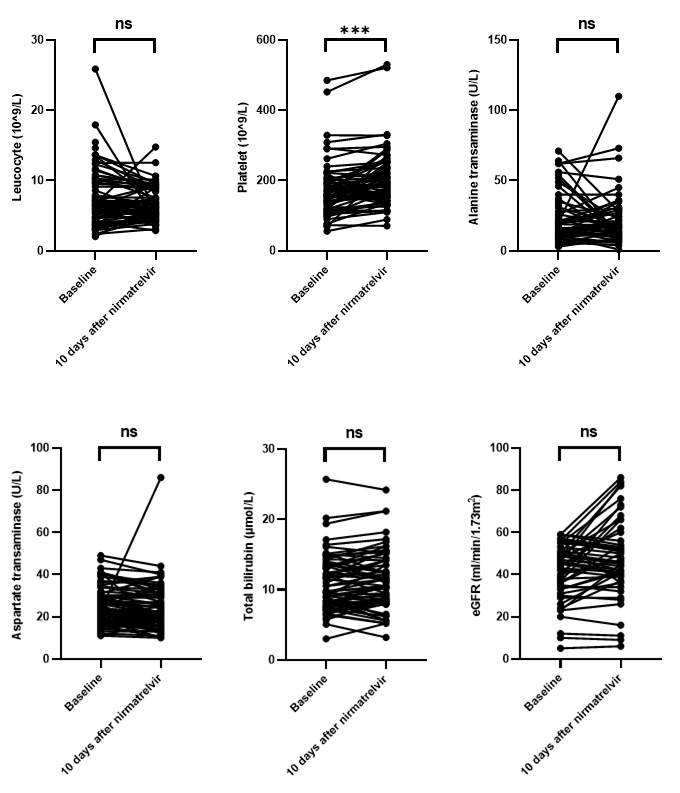

Supplement: Supplementary file 1 [file Table1.docx]
